# Dissemination feature based on PET/CT is a risk factor for diffuse large B cell lymphoma patients outcome

**DOI:** 10.1186/s12885-023-11333-z

**Published:** 2023-11-29

**Authors:** Fei Wang, Silu Cui, Luo Lu, Xiaoliang Shao, Feng Yan, Yaqi Liu, Bai He, Jianfeng Wang, Yang Cao, Yanhua Yue, Yuetao Wang, Weiying Gu

**Affiliations:** 1 Department of Hematology, The First People’s Hospital of Changzhou, The Third Affiliated Hospital of Soochow University, Changzhou, Jiangsu China; 2 Department of Nuclear Medicine, The First People’s Hospital of Changzhou, The Third Affiliated Hospital of Soochow University, Changzhou, Jiangsu China

**Keywords:** Dissemination, Distance, PET/CT, Diffuse large B-cell lymphoma, Prognosis

## Abstract

**Background:**

^18^F-FDG PET/CT provides precise information about dissemination of lymphoma lesions. Dmax, defined as distance between the two lesions that were farthest apart by PET/CT, was found to be a promising predictor of Diffuse large B-cell lymphoma (DLBCL) outcome in a small size of clinical trial data. We analyzed the impact of Dmax on the outcome of a large real-world DLBCL cohort.

**Methods:**

Data of newly diagnosed DLBCL at the Third Affiliated Hospital of Soochow University were retrospectively collected. Baseline Dmax, clinical data and survival information were recorded. A metabolic parameter, metabolic bulk volume (MBV), was also measured to verify the independent impact of Dmax.

**Results:**

Optimal cut-off values for Dmax and MBV were 45.34 cm and 21.65 cm^3^. With a median follow-up of 32 months, Dmax significantly impacted progression-free survival (PFS) and overall survival (OS) in 253 DLBCL patients. For Dmax^low^ and Dmax^high^ groups, estimated 3-year OS were 87.0% and 53.8% (*p* < 0.001), while 3-year PFS were 77.3% and 37.3% (*p* < 0.001). And for MBV^low^ and MBV^high^groups, 3-year OS were 84.5% and 58.8% (*p* < 0.001), and 3-year PFS were 68.7% and 50.4% (*p* = 0.003). Multivariate analysis identified Dmax and Eastern Cooperative Oncology Group performance status (ECOG PS) independently associated with PFS and OS, while MBV only independently associated with OS. A Dmax revised prognostic index (DRPI) combining Dmax and ECOG PS identified an ultra-risk DLBCL population with 3-year PFS of 31.7% and 3-year OS of 38.5%. The area under the curve (AUC) showed that this model performed better than International prognostic Index (IPI).

**Conclusion:**

Dmax is a new and promising indicator to investigate dissemination of lymphoma lesions associated with the outcome of DLBCL. It significantly contributes to stratification of patients with disparate outcomes.

**Trial registration:**

This research has been retrospectively registered in the Ethics Committee institutional of the Third Affiliated Hospital of Soochow University, and the registration number was approval No. 155 (approved date: 31 May 2022).

**Supplementary Information:**

The online version contains supplementary material available at 10.1186/s12885-023-11333-z.

## Background

Diffuse large B cell lymphoma (DLBCL) represents almost 30% of non-Hodgkin lymphoma (NHL) [1], which is responsible for 544,000 new cases and 260,000 deaths in 2020 [2]. Despite the remarkable success of immune-chemotherapy of RCHOP (Rituximab, cyclophosphamide, doxorubicin, vincristine, and prednisone), outcomes remain variable among patients. Long time survival has been achieved in approximately two-thirds of the patients [3], but prognosis is dismal for patients failed the first-line treatment, with 7% complete response and 6 months overall survival (OS) [4].The International prognostic Index (IPI), consisting of age, Eastern Cooperative Oncology Group performance status (ECOG PS), Ann Arbor stage, serum lactate dehydrogenase (LDH), and extranodal involvement, was induced in 1993 [5]. Since then, massive attempts have been made to adjust the scoring system to better delineate patients with inferior outcome [6–8]. Ultimately, National Comprehensive Cancer Network -IPI (NCCN-IPI), with the same clinical parameters but detailed grading of each risk factor, demonstrated the best performance to estimate the highest and lowest risk groups. Nevertheless, none of these scoring systems can identify a subgroup with less than 50% of long-term survival [9].

^18^F-labeled fluorodeoxyglucose positron emission tomography with computed tomography (^18^F-FDG PET/CT) is highly sensitive for detecting DLBCL lesions. By PET/CT, more accurate methods to identify patients with early treatment failure have been recognized. Mounting evidence ensured the irreplaceable role of PET/CT for staging in a variety of NHLs, including DLBCL, making it an essential part in DLBCL patients management [10]. PET/CT provided accurate disease mapping with distinct lesions across the whole body, which of the most concern are the metabolic features of lymphoma. The most commonly used parameter is the standardized uptake value (SUV) at sites of disease [11]. Other metabolic parameters, including metabolic tumor volume (MTV) and metabolic bulk volume (MBV), were also recognized as independent predictors for DLBCL prognosis [12–14]. MBV was proved to be significantly associated with MTV [14], and different studies demonstrated its satisfactory performance in predicting survival not inferior sometimes even superior than MTV [13–15]. Considering diffusion of disease lesions a challenge for radiomic analysis of lymphoma, the accurate prediction of outcome and relatively easy access made MBV a potentially more valuable marker to assess metabolic feature of lymphoma. Besides the metabolic uptake of the disease, the dissemination of the disease lesions presented in PET/CT was also an ignorable information. Recently, a new PET/CT metric describing tumor dissemination defined as the distance between the 2 farthest lesions, named Dmax [16], was proved to be a promising predictor associated with inferior outcome of DLBCL [11, 16]. As a semi-quantitative dissemination indicator, Dmax presented strong prognostic value independent of IPI and MTV in elderly patients ranging from 60 to 80 [11]. And to date, the clinical value of Dmax and the correlation with the clinical characteristics in a large number of DLBCL cohort across whole age spectrum has not been fully explored.

Here, we develop a new approach based on 3-dimensional imaging to detect Dmax, and sought to investigate the dissemination presented by Dmax in a real-world data to evaluate whether baseline Dmax demonstrated prognostic value in a large retrospective cohort of DLBCL, and whether it may improve the prediction of prognosis in DLBCL patients, especially for identification of those high-risk patients. And meanwhile, we measured the metabolic volume of the largest lesion, MBV, to evaluate its impact on the outcome of DLBCL patients as a potentially surrogate indicator determining the metabolic feature. Thereby we try a new model combining PET/CT indicators and clinical predictors to better stratify DLBCL patients, especially for the ultra-risk population.

## Methods

### Study population and clinical data

Data of newly diagnosed DLBCL patients at the Third Affiliated Hospital of Soochow University’s institutional DLBCL cases between May 2012 and November 2021 were retrospectively collected. PET/CT was not practiced as routine examination before 2015 in our institution, thus there were only 11 patients included between 2012 and 2015, who were excluded from the analysis. Meanwhile, more patients underwent PET/CT baseline assessment over time. Pathological diagnosis was strictly based on the morphological and immunohistological criteria of the World Health Organization classification, and all special DLBCL subtypes, including primary cutaneous B-cell lymphoma, primary mediastinal large B-cell lymphoma, primary DLBCL of the central nervous system and transformed DLBCL, were excluded.

This study was performed in accordance with the Declaration of Helsinki and approved by the Ethics Committee institutional of the Third Affiliated Hospital of Soochow University. The requirement for written patient consent was waived as a retrospective study. All identifiers were removed after the completion of our analyses to protect patient privacy. Baseline clinical characteristics including age, gender, LDH, B symptoms, ECOG PS and Ann Arbor stage, Hans classification, Bulky disease (≥ 7.5 cm), were recorded during admission. To allow for the measurement of baseline Dmax, only patients with a baseline PET/CT and more than one suspicious lymphoma lesion were included.

### Baseline Dmax and MBV and Quality control of ^18^F-FDG PET/CT scans

Imaging was performed with a 64-MDCT PET/CT scanner (Biograph mCT, Siemens Healthcare). The imaging agent was FDG (radiochemical purity, > 95%). All patients fasted for at least 8 h before the PET/CT examination, and their serum glucose level was less than 11 mmol/L. After intravenous injection of FDG at 4.44 MBq/kg, the patients rested in a quiet, warm, sheltered environment for 45–60 min. After urinating, the patients underwent PET/CT. Automatic exposure control (CARE Dose 4D, Siemens Healthcare) was used for CT. Tube current was automatically adjusted according to the body’s shape, anatomic structure and tissue density. Tube voltage was 100 kV; pitch, 0.8; single layer rotation time, 0.5 s; slice thickness, 3 mm. The PET mode was 3D. The scan range was skull base to upper femur, and the acquisition time was 2 min per bed. Images were reconstructed (SyngoTrueD system, Siemens Healthcare) to produce cross-sectional, coronal, and sagittal tomographic images and 3D projection images.

**Fig. 1:**
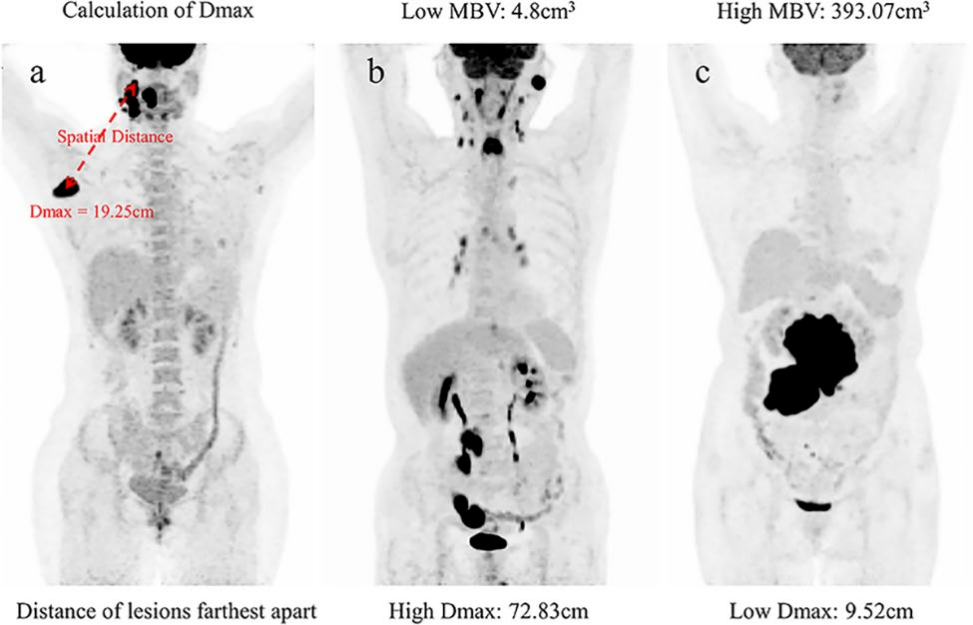
Measurement of Dmax and differences of Dmax and MBV. (**a**) Using the 3-dimensional imaging reconstructed, Dmax is defined as the spatial distance between the two lesions that are farthest apart. (**b**) Example patient with high Dmax and low MBV, experienced early relapsed and death. (**c**) Example patient with low Dmax and high MBV, remained remission at follow-up time

PET/CT images were viewed on a post-processing video display provided by the equipment manufacturer. All measurement was independetly carried out by two nuclear medicine physicians by images 3D reconstructed. Regional volumes were identified automatically by the software, and then checked visually to confirm pathological lesions. For the lesions with controversial values, it is determined through discussion in combination with the medical history. Neither nuclear medicine physician had any information about the patient’s clinical prognosis. The dissemination feature, Dmax, was extracted as suggested by Cottereau et al. [16], defined as the distance between the two lesions that were farthest apart. The nuclear medicine physician selected each hypermetabolic lesion by clicking on its projection using a graphical user interface. Through three-dimensional reconstruction, the center of the lesion was automatically determined, and the distances between all paired lesions were directly obtained by the system, of which the largest distance was recorded as Dmax (Fig. 1a).

In addition, MBV was measured semi-automatically with an SUV based automatic contour program using reconstructed images (Syngo TrueD System Siemens Healthcare) and defined as the metabolic volume of the largest lesion as previously reported [13, 14]. Nuclear medicine physicians clicked on the projection of each hypermetabolic lesion in the graphical user interface, and the system automatically generated the contour around the target lesion within the boundary and defined metabolic volume with voxel within the contour boundary presenting a 41% SUVmax threshold. The maximum value was recorded as MBV (Supporting information Fig. S1). Examples of PET images (maximum-intensity projections) of patients with differences Dmax and MBV were shown in Fig. 1, illustrating the different feature with high Dmax but low MBV (Fig. 1b) and low Dmax but high MBV (Fig. 1c).

Data from the two nuclear medicine physicians were tested by intraclass correlation efficient (ICC), showing fairly high concordance between their measurements for both MBV (0.992, p < 0.001) and Dmax (0.989, p < 0.001) respectively.

### Follow-up and endpoints

All patients were followed up until March 31, 2022 or the death of patients through making telephone calls and rechecking medical records. OS was defined as the interval of time between pathological diagnosis and death from any cause or the last follow-up, and PFS as the interval of time between pathological diagnosis and progression of the tumor for any aspect,death from any reason, or the last follow-up. The survival status of all patients was confirmed through death records or telephone call to immediate family (in the case of patients death during the follow-up) or to the patients themselves.

### Statistical analysis

Categorical variables were recorded as numbers (percentages) and continuous variables as and median (interquartile range, IQR). All the individual factors of IPI were categorized by conventional criteria. χ^2^ test were used to analyze the differences for clinical factors. Dmax and MBV were transformed into a categorical variable by MaxStat analysis (titled as Maximally Selected Rank Statistics). The univariate association between PFS and OS were analyzed by Cox proportional hazard model. The Kaplan–Meier method was used to calculate survival curves. The variables with significance in univariable analysis were kept in the multivariate analysis. All the statistical tests were two-sided, with the statistical significance set at *p* < 0 0.05.

All data were calculated by IBM SPSS 21.0 (IBM Inc., Armonk, USA), R software (version 4.0.3; http://www.Rproject.org) and Stata version 15.0.

## Results

**Fig. 2:**
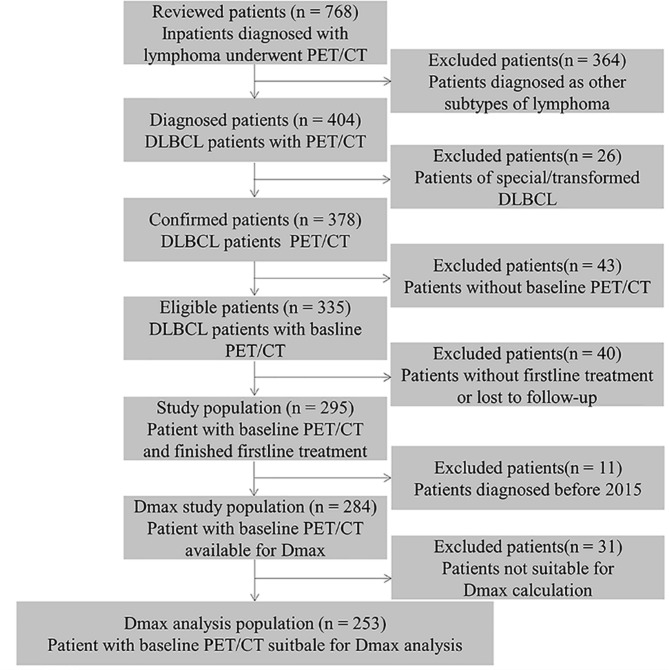
Flowchart of patients by Dmax analysis PET/CT, positron emission tomography/computed tomography; DLBCL, Diffuse large B cell lymphoma

A total of 284 patients were retrospectively collected with baseline PET/CT scan before treatment engagement. Of them, 253 patients have more than one lesion to determine Dmax (Fig. 2). The mean baseline Dmax was 37.37 cm across the whole cohort (median 30.83 cm, range 2.47–93.13), while the mean MBV was 106.71 cm^3^ (median 18.25 cm^3^, range 0.50-597.91).

Dmax was significantly associated with PFS (HR 1.019, 95% CI: 1.012–1.027, *p* < 0.001) and OS (HR 1.023, 95 CI: 1.013–1.033, *p* < 0.001) of DLBCL patients as a continuous value. Similar results of PFS (HR 1.002, 95% CI 1.001–1.004, p = 0.005) and OS (HR 1.003, 95% CI 1.001–1.005, p < 0.001) were also reported for MBV as a continuous value. Multivariable analysis further identified Dmax as an independent factor for both PFS (HR 1.011, 95% CI 1.001–1.022, *p* = 0.026) and OS (HR 1.016, 95% CI 1.003–1.029, *p* = 0.017) when all the clinical indicators and MBV were included in the model. Meanwhile, MBV presented as an independent predictor for OS (HR 1.002, 95% CI 1.000-1.005, *p* = 0.029) but not PFS (HR 1.001, 95% CI 0.999–1.003, p = 0.160) (Table S1).

**Fig. 3:**
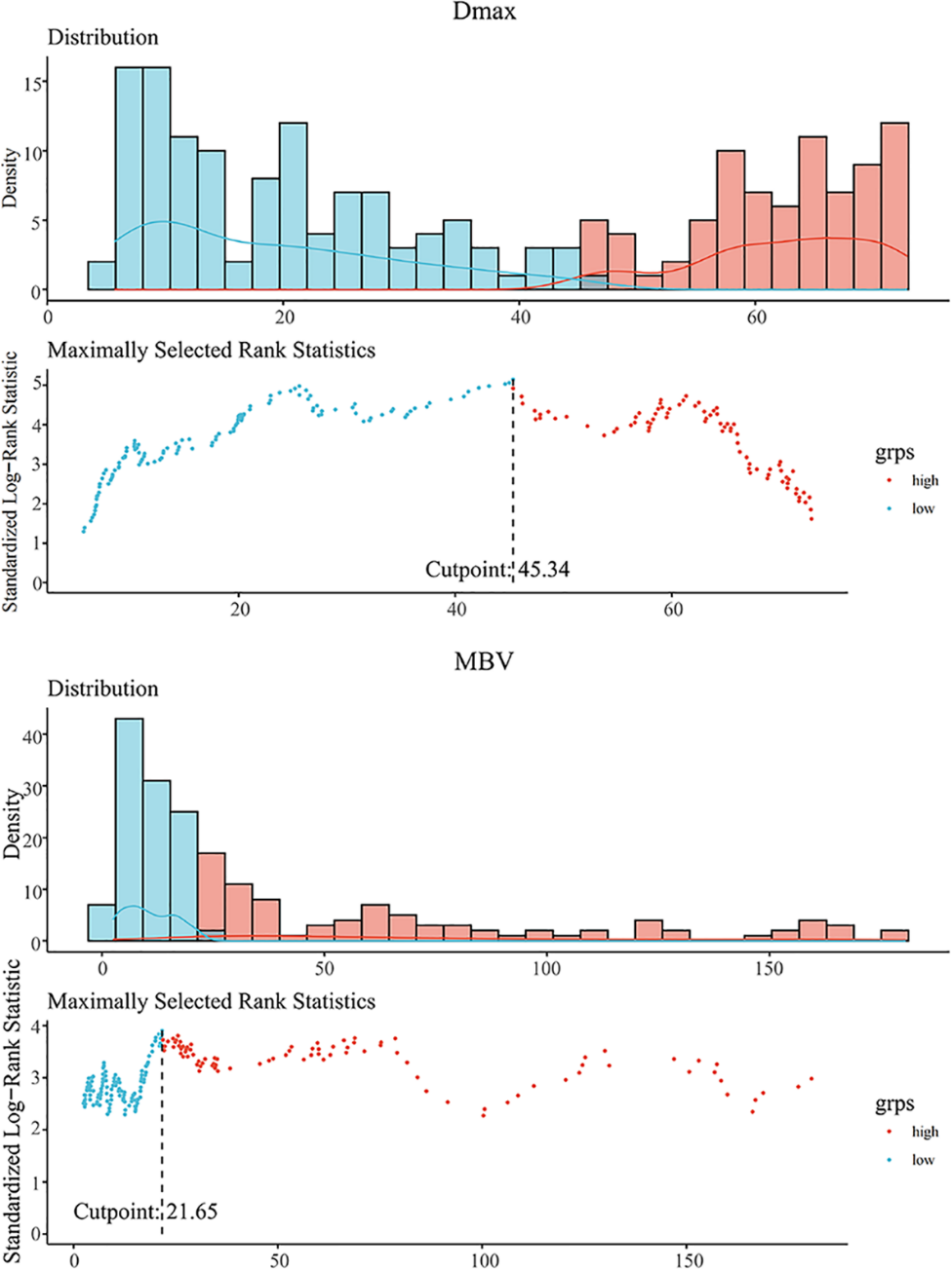
Cut-off points of Dmax and MBV defined by using maximally selected log-rank statistics The estimated optimal cut-off point of Dmax was 45.34 cm (**A**), and optimal cut-off point of MBV was 21.65cm^3^ (**B**) MBV, metabolic bulk volume

According to the maximal chi-square method, 45.34 cm and 21.65 cm^3^ were the optimal cut-off values respectively for the Dmax and MBV that distinguished between different prognostic groups for OS most effectively (Table 1; Fig. 3). Applying these results, patients were respectively divided into two groups of Dmax and MBV as for those greater than the cut-off values refined as Dmax^high^ or MBV^high^ group. The analysis besides were all based on the new definition.

**Table 1:** Optimal cut-off of Dmax and MBV for OS based on maximal chi-square method

MAXSTAT	Dmax	MBV
Chi-square value	5.149	3.903
Optimal cut-off	45.34	21.65
*P* value	< 0.001	< 0.001

MBV, metabolic bulk volume

### Clinical characteristics and Dmax of DLBCL patients

Baseline demographic and clinical characteristics are shown in Table 2. During a median follow-up of 32 months, 94 (37.2%) patients experienced overt disease progression, and 64 (25.3%) of them ultimately succumbed to the disease. Median PFS and OS was 88 months and not reached respectively. Estimated 3-year OS and 3-year PFS was 72.8% and 60.4% for the whole population. Of the entire cohort, 106 (41.9%) patients were clarified as Dmax^high^ group, and 115 (45.5%) as MBV^high^ group. Meanwhile, distinguished Dmax and MBV have poor correlation considering the fairly low correlation coefficient (Supporting information Table S2). Median age was 65 years (range13-91), and no significant difference of Dmax was claimed between with the elder (> 60) and younger (≤ 60) patients. Staging were analyzed based on two different methods, verifying 158 (62.5%) advanced stage (stage III/IV) based on Ann Arbor stage, 166 (65.6%) advanced stage (stage III/IV) based on Lugano stage. Both were shown associated with higher Dmax. The other two individual IPI factors including elevated LDH (> 250 U/L), extranodal involvement (> 1) were also significantly associated with Dmax, subsequently yielded the association of higher IPI score with high Dmax.

**Table 2:** Baseline characteristics for the whole population and stratified according to pretreatment Dmax with 45.32 cm cutoff

	Dmax ≤ 45.32 (147)	Dmax > 45.32 (106)	*P-*value
Age, year, (range)	61 (18–91)		0.281
Age ≤ 60	64 (43.5%)	39(36.8%)	
Age > 60	83 (56.5%)	67 (63.2%)	
Gender			0.905
Male	76 (51.3%)	54 (50.0%)	
Female	71 (48.7%)	52 (50.0%)	
Ann Arbor Stage			**< 0.001**
Stage II	93 (64.3%)	2 (1.8%)	
Stage III/IV	54 (35.7%)	104 (98.2%)	
Lugano Stage			**< 0.001**
Stage II	86(59.7%)	1 (0.9%)	
Stage III/IV	61 (40.3%)	105 (99.1%)	
LDH (U/L)			
Normal (≤ 250)	96 (66.2%)	33 (30.0%)	**< 0.001**
High (> 250)	51 (33.8%)	73 (70.0%)	
Extranodal site			**< 0.001**
0–1	107 (73.4%)	36 (33.6%)	
> 1	40 (26.6%)	70 (66.4%)	
ECOG PS			**< 0.001**
0–1	115 (77.3%)	60 (55.5%)	
≥ 2	32 (22.7%)	46 (44.5%)	
Bulky disease			**0.006**
< 7.5 cm	117 (79.9%)	68 (63.6%)	
≥ 7.5 cm	30 (20.1%)	38 (36.4%)	
COO			0.644
GCB	95 (69.0%)	66 (67.3%)	
Non-GCB	43 (31.0%)	34 (32.7%)	
IPI score			**< 0.001**
0–2	105 (72.1%)	23 (21.8%)	
> 3	42 (27.9%)	83 (78.2%)	
NCCN-IPI			**< 0.001**
Low intermediate (0–3)	92 (63.0%)	16 (14.5%)	
Intermediate high and high (≥ 4)	55 (37.0%)	90 (85.5%)	

LDH, serum lactate dehydrogenase; ECOG PS, Eastern Cooperative Oncology Group performance status; COO, cell-of-origin; GCB, germinal center B-cell; IPI, International prognostic Index; NCCN, National Comprehensive Cancer Network

### Univariable analysis

Univariable analysis for prognosis of all the clinical factors in the entire cohort was shown in Table 3. The five individual IPI indexes were identified as being prognostic for inferior PFS and OS: elderly age, elevated LDH level, advanced stage, etxtranodal lesions > 1 and ECOG PS ≥ 2. Patients with high Dmax or MBV also experienced high risk of progression and death. PFS and OS did not differ differently by Cell-of-origin (COO) determined by immunohistochemistry.

**Table 3:** Prognostic impact of Dmax on PFS and OS in the entire cohort

Variables	PFS						
	Univariable analysis				Multivariable analysis		
	HR	95% CI	*p* value		HR	95% CI	*p* value
Age	1.017	1.001–1.033	0.040		1.003	0.986–1.020	0.738
Advanced Stage (Ann Arbor)	3.827	2.198–6.666	< 0.001		2.046	1.022–4.098	0.043
Advanced Stage (Lugano)^1^	3.595	2.035–6.353	< 0.001				
LDH ˃250	1.764	1.164–2.673	0.007		0.804	0.475–1.363	0.419
Extranodal sites > 1	1.988	1.318–2.998	0.001		1.070	0.674–1.699	0.773
ECOG PS ≥ 2	2.378	1.577–3.587	< 0.001		1.803	1.133–2.870	0.013
Dmax (> 45.34 cm)	3.068	2.002–4.701	< 0.001		1.821	1.090–3.042	0.022
MBV (> 21.65cm^3^)	1.828	1.213–2.754	0.004		1.435	0.881–2.337	0.147
Bulky (≥ 7.5 cm)	1.739	1.136–2.662	0.011		1.130	0.682–1.873	0.634
COO (nonGCB/GCB)	1.114	0.718–1.729	0.630				
Variables	OS						
	Univariable analysis				Multivariable analysis		
	HR	95% CI	*p* value		HR	95% CI	*p* value
Age	1.040	1.018–1.062	< 0.001		1.029	1.005–1.055	0.020
Advanced Stage (Ann Arbor)	4.731	2.253–9.936	< 0.001		2.048	0.824–5.090	0.123
Advanced Stage (Lugano)^1^	4.119	1.963–8.642	< 0.001				
LDH ˃250	2.394	1.420–4.037	0.001		0.926	0.486–1.763	0.814
Extranodal sites > 1	1.820	1.110–3.985	0.018		0.736	0.424–1.275	0.274
ECOG PS ≥ 2	3.593	2.191–5.891	< 0.001		2.348	1.346–4.099	0.003
Dmax (> 45.34 cm)	3.765	2.200-6.443	< 0.001		2.076	1.093–3.941	0.026
MBV (> 21.65cm^3^)	2.703	1.611–4.533	< 0.001		2.116	1.160–3.857	0.014
Bulky (≥ 7.5 cm)	2.078	1.259–3.428	0.004		1.021	0.564–1.847	0.946
COO (nonGCB/GCB)	1.068	0.626–1.823	0.809				

^1, 2^Multivariable analysis only included stage based on Ann Arbor system

CI, confidence interval; COO, cell-of-origin; ECOG PS, Eastern Cooperative Oncology Group performance status; HR, Hazard Rate; LDH, serum lactate dehydrogenase; MBV, metabolic bulk volume; OS, overall survival; PFS, progression-free survival

**Fig. 4:**
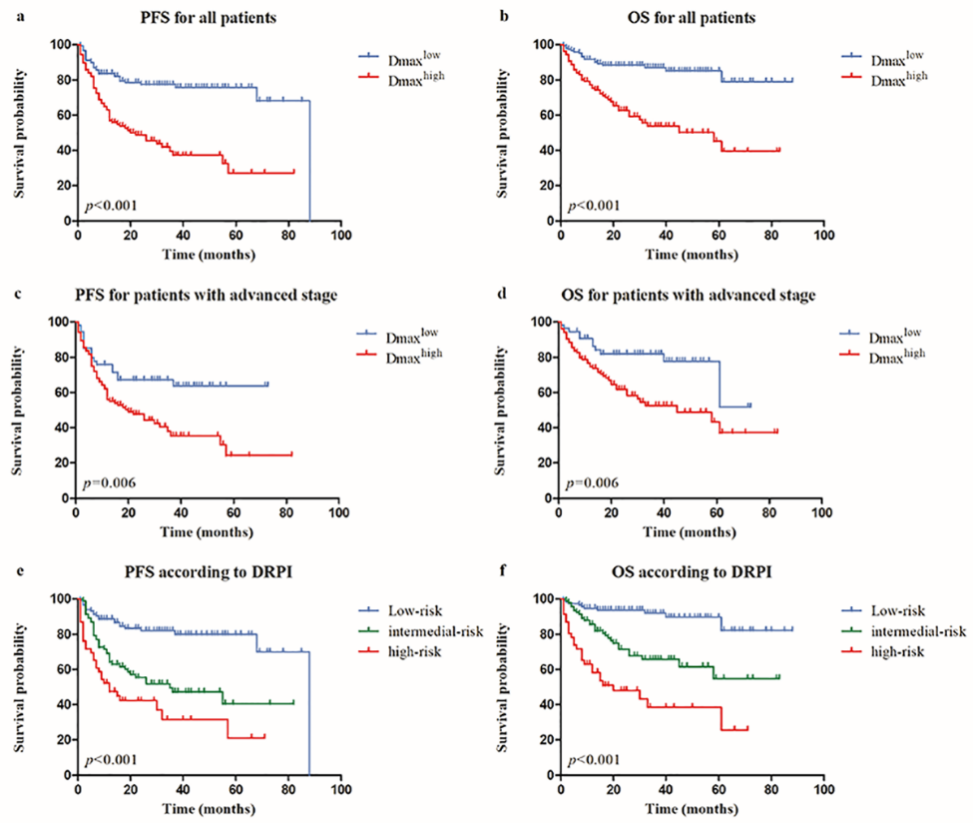
Kaplan–Meier estimates of PFS and OS according to baseline Dmax and risk stratification (**A**) PFS for all DLBCL patients, (**B**) OS for all DLBCL patients, (**C**) PFS in patients with advanced stage based on Ann Arbor system, (**D**) OS in patients with advanced stage based on Ann Arbor system, (**E**) PFS for patients according to DRPI, (**F**) OS for patients according to DRPI. DLCBL, Diffuse large B cell lymphoma; DRPI, Dmax revised prognostic index; OS, overall survival; PFS, progression-free survival

For the 106 patients with high Dmax, median PFS was 22 months (95% CI 8.137–35.863), and median OS was 58 months (95% CI 31.116–84.884), and were significantly worse than the patients presented with low Dmax, whose median PFS and OS were 88 months and not reached during the follow-up time. For Dmax^low^ and Dmax^high^ group, estimated 3-year OS were 87.0% and 53.8%, while 3-year PFS were 77.3% and 37.3%, respectively (Fig. 4a, b). Meanwhile, 3y-OS was 84.5% and 58.8%, 3y-PFS was 68.7% and 50.4% for MBV^low^ and MBV^high^ patients, respectively.

Based on Ann Arbor system, 95 patients were staged as stage II, and only 1 case was defined as Dmax^high^. This patient did not experience any disease events during the following-up time. For the 158 patients of advanced stage, 104 of them are grouped as Dmax^high^, whose outcome was significantly worse than the other 54 Dmax^low^ patients. Median PFS was 20 months (95% CI 8.005–31.995) and not reached, and median OS was 45 months (95% CI 17.973–72.027) and not reached, and estimated 3-year PFS was 63.8% and 35.4%, 3-year OS was 81.9% and 52.6% for Dmax^high^ and Dmax^low^ group patients of advanced stage, respectively (Fig. 4c, d).

### Multivariable analysis

Combining all the factors potentially associated with PFS or OS, the multivariable analysis ensured the independent significance of Dmax, as well as ECOG PS on both PFS and OS (Table 3). Age and high MBV was an independent risk factor for OS but not for PFS, and advanced Ann Arbor stage was an independent inferior factor only for PFS. Besides, when included Lugano stage instead of Ann Arbor stage, Dmax remained its independent impact on PFS and OS, but Lugano stage seemed did not have independent significance on both PFS and OS (Supporting information Table S3). For the 158 patients presented as advanced stage of Ann Arbor, Dmax and ECOG PS were the remaining two independent risk factors for PFS and OS, while MBV only impacted significantly on OS (Table 4).

**Table 4:** Multivariable analysis of PFS and OS for advanced Ann Arbor stage patients

Variables		PFS				OS	
	HR	95%CI	*p* value		HR	95%CI	*p* value
Dmax (> 45.34 cm)	2.018	1.170–3.481	0.012		2.260	1.150–4.442	0.018
MBV (> 21.65cm^3^)	1.704	0.991–2.932	0.054		2.245	1.169–4.313	0.015
Age	0.992	0.975–1.010	0.399		1.017	0.991–1.043	0.197
Extranodal sites > 1	1.186	0.730–1.926	0.492		0.816	0.459–1.449	0.488
LDH > 250	0.645	0.360–1.155	0.140		0.764	0.381–1.531	0.448
ECOG PS ≥ 2	2.078	1.262–3.421	0.004		2.762	1.523–5.011	0.001
Bulky (≥ 7.5 cm)	1.102	0.644–1.886	0.724		1.015	0.544–1.894	0.962

CI, confidence interval; ECOG PS, Eastern Cooperative Oncology Group performance status; HR, Hazard Rate; LDH, serum lactate dehydrogenase; MBV, metabolic bulk volume; OS, overall survival; PFS, progression-free survival

IPI and Dmax were all independently significant risk factors for PFS and OS, and Dmax was shown to be a strong independent risk factor independent of IPI score (Supporting information Table S4).

### Combination of baseline Dmax optimizes prediction of patient outcomes 

**Fig. 5:**
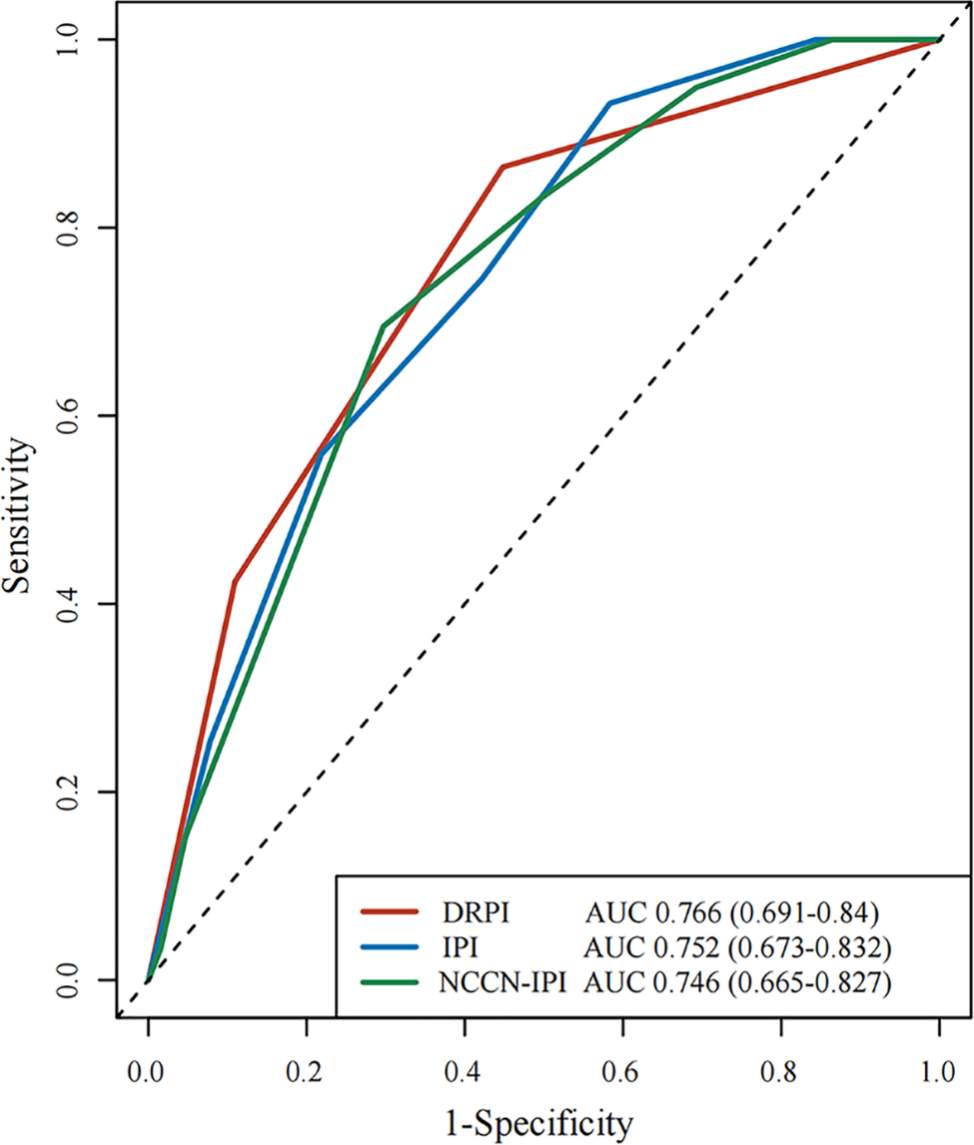
Receiver operating characteristic curves for OS for DRPI, IPI and NCCN-IPI prediction models AUC, area under the curve; DRPI, Dmax revised prognostic index; IPI, International prognostic Index; NCCN, National Comprehensive Cancer Network, OS, overall survival

Considering the independent significant impact of ECOG PS and Dmax for PFS and OS, a new prediction model defined as Dmax revised prognostic index (DRPI) was established based on these two factors. One point was recorded for each factor of ECOG PS ≥ 2 and high Dmax. Patients were divided into three groups: low-risk group with 0 point, intermediate-risk groupwith 1 point and high-risk group with 2 points. Significant differences was observed between the different groups, which showed estimated 3-year PFS was 82.0%, 47.2% and 31.7%, and 3-year OS was 91.9%, 65.8% and 38.5%, respectively (Fig. 4e, f). The DRPI was significantly associated with outcome, yielding an area under the curve (AUC) of 76.6%, which indicated a better performance than both IPI and NCCN-IPI in this group of DLBCL patients (Fig. 5).

## Discussion

The current research revealed the strong and significant prognostic value for DLBCL patients of baseline Dmax, a semi-quantitative dissemination parameter calculated by PET/CT. Prognosis of patients with high baseline Dmax was very frustrating, whose 3y-PFS and 3y-OS were only 37.3% and 53.8%, respectively. Accounting for more than one-third of all DLBCL patients, Dmax^high^ patients presented with a more than 30-point reduction of both 3-year PFS and OS compared with the Dmax^low^ patients. The impact maintained irrespective of the Stage based on both Ann Arbor and Lugano system. Multivariable analysis confirmed the independent prognostic value of Dmax, as well as ECOG PS. A new clinical model based on these two factors also presented better performance than IPI and NCCN-IPI in predicting OS of DLBCL patients.

The heterogeneity of DLBCL still posed a great threat for the treatment and outcome of the patients. DLBCL typically had no primary sites and could affect any part of the whole body. Until now, there were no practical clinical indicators that could precisely describe the diffusion degree of the disease. The only clinical indicator implicating the dissemination to certain extent was stage of the disease, which was generally carried out based on Ann Arbor system. This modality of anatomic stage has been adopted for over half a century, presuming the equivalent prognosis for patients with simultaneous involvement of upper and (or) lower diaphragm [17]. In 2014, the system was revised with more attention to extranodal lesions [18]. Whether as a continuous or categorical variable, Dmax presented as an independent prognostic factor for both PFS and OS of DLBCL patients, while Ann Arbor stage only independently impact PFS not OS. Meanwhile, Dmax was still an independent predictor for PFS and OS even for patients of advanced stage of both systems. For the 258 patients presenting more than one lesions in our study, only eight patients altered from early stage based on Ann Arbor system to advanced stage identified by Lugano stratification. Lugano system was not independently associated with neither PFS nor OS. This also implicated that the extent of disease dissemination has a greater impact on the disease prognosis than the extranodal involvements considering DLBCL as a systemic disease.

PET/CT is a routine and currently state-of-the-art modality for detecting lesions of DLCBL without omission owing to its high sensitivity, making it possible to develop a more accurate parameter to describe disease dissemination. The application of PET/CT led to changes in stage for 10-30% patients of lymphoma, usually upstaging, [19] resulting in alterations in clinical management of 3-25% patients [20]. Improved delineation of disease involvement provided by PET/CT, even for early stage patients, suggested the magnitude of change exceeds stage migration. The tumor burden and metabolic characteristics of lymphoma measured by PET/CT have long been realized as prognostic factors for DLBCL. MTV was independent prognostic value for the disease despite variations in measurement methods and cut-off values in massive reports, [21–24] and was proved to have linear spline relationship with survival of DLBCL patients [12]. MBV was also pronounced as independent predictor for PFS and OS, and can serve as a good substitute for MTV [13, 14]. MBV was firstly reported as MTV of the largest tumor lesion, and was proved to be associated with both PFS and OS of DLBCL. When combined with MTV, ECOG PS and bulky disease, MBV was proved to be independently associated with OS, while MTV only with PFS [13]. MBV was significantly correlated with MTV, and for patients discordance between the MBV and MTV, MBV seemed to be more predictable for PFS. Another study declared the independent impact of MBV on both PFS and OS of stage II/III DLBCL patients [15].

In our research, patients with low MBV presented significant superior PFS and OS. Although subsequent evidence did not identify MBV as an independent prognostic factor for PFS, it still revealed MBV as an independent predictor of OS in DLBCL patients. Given the difficulty of MTV measurements in clinical practice, MBV remained valuable in lymphoma as a conventionally obtainable volumetric FDG parameter. Further researches would be needed for the exploration of MBV and MTV, as well as other metabolic parameters provided by PET/CT. Meanwhile, the bulky disease did not show independent impact on both PFS and OS, further clarified the metabolic feature provided by PET/CT more valuable for clinical outcome.

Dmax was firstly identified by Cottereau et al. in a small size of 95 DLBCL patients from LNH073B study. Compared with other three different dissemination parameters, including Dmax_bulk_ (defined as the maximal distance between the largest lesion and any other lesion), SPREAD_bulk_ (the sum of the distances of the bulky lesion from all other lesions) and SPREAD_patient_ (the largest value, over all lesions, of the sum of the distances from a lesion to all the others), Dmax was the only independent predictor for both PFS and OS [16]. The author further investigated Dmax in REMARC clinical trial cohort containing 301 DLBCL patients aged 60–80 years and verified its independent predictive value in elderly patients. In our research, Dmax was a strong predictor for the outcome of DLBCL, consistent with their results. Further indicating Dmax as a promising parameter to assess the dissemination of lymphoma lesions. Impressively, high standardized Dmax combined with high MTV and ECOG PS revealed to be associated with high risk of CNS relapse for the elderly [11]. Since then, different retrospective researches have demonstrated the promising value of Dmax for lymphoma. A systematic review claimed the dissemination of disease assessed by Dmax significantly associated with the outcome of many different types of lymphoma, including HL, DLBCL, Peripheral T-cell Lymphomas (PTCL) and Angioimmunoblastic T-cell lymphoma (AITL) [25]. As a simple and intuitive indicator, Dmax is a very easilly assessed dimensional feature, and is less influenced by “technical” features. Combination of Dmax and different radiomics and clinical makers released significant improvement of patients risk-stratification of lymphoma [25]. Combination of MTV and Dmax could recognize the high-risk patients presenting 4y-PFS and 4y-OS of 41% and 66% respectively in previous report [11], while in our research, combination of the two independent predictors of Dmax and ECOG PS, could help us identify ultra-risk patients with 3y-PFS and 3y-OS of only 31.7% and 38.5%. Nevertheless, the predictive value and the association of Dmax with other clinical predictors has not been explored in a large DLBCL cohort of all ages. Another model consisted of Dmax_bulk_ and the natural logarithms of MTV and of SUVpeak presented better performance than IPI to predict 2-year time to progression (TTP) [26]. The metabolic feature of DLBCL defined by MTV has poor correlation with Dmax [16], consistent with our results of MBV and Dmax, indicating Dmax as a unique and predictive characteristic of DLBCL.

This is the first large-scale study to investigate the predictive value of dissemination feature in DLBCL at all ages, which also combined it with currently used clinical predictors, making it the most comprehensive study so far. Meanwhile, the two staging systems were all included in our research to further confirm the prognostic value of Dmax as an indicator of disease dissemination characteristics. Moreover, to determine the exact distance of Dmax, new approach based on 3D reconstructed was applied here, providing more direct and precise method to evaluate the parameter.

The international index has been practiced for nearly thirty years, and emergence of new technologies have notably improved the understanding of the disease. As PET/CT has already been widely used as a routine examination during clinical management of DLBCL, combination of the extensive and new disease information provided by PET/CT with the clinical data brought dramatic improvement to the risk stratification and treatment management. A new model established here on two independent predictors, including Dmax and ECOG PS, performed better prediction for OS than IPI and NCCN-IPI, paving the way for future exploration of new stratification. This new model divided patients into three distinct risk groups with 3-year OS gapping more than 25%. For the patients in the ultra-risk group, manifesting high Dmax more than 45.34 cm, ECOG PS ≥ 2, nearly two thirds of them ultimately died within 3 years, corresponding with 5-year OS for high-risk group in IPI of 43% and NCCN-IPI of 38% [7].

As for the biomarker, chemokine CXCR4 plays an essential role in tumor dissemination and progression [27], and is associated with inferior outcome [28, 29]. In vivo models, decreased lymphoma dissemination was pronounced associated with prolonged animal survival via diverse mechanisms mainly through inhibition of Human germinal center-associated lymphoma (HGAL) [30]. Wide application of PET/CT makes it feasible to evaluate Dmax in clinical practice, and it is rather desirable for further investigation for the dissemination characteristics of the disease of both clinical manifestations and biological markers, which might help us to further comprehend the essentials of the disease.

Our research had some inevitable limitations as a single-center retrospective study. As PET/CT was not mandatory at baseline evaluation, some biases of the patients are not inevitable in patients’ economic status and physician preference, resulting in some selection bias of the patients. The follow-up time was relatively short considering the long survival of the disease, and the relatively small number of patients diagnosed before 2015 may further impact the results of survival analysis. The results lack external validation to confirm its value, and also need to be validated in a larger population from prospective multi-center studies.

## Conclusion

In conclusion, the semi-quantitative factor of disease dissemination Dmax, is a robust and independent predictor of survival outcomes for DLBCL. High Dmax at baseline was significantly associated with inferior PFS and OS in patients. Baseline Dmax combined with ECOG PS could improve risk stratification for DLBCL patients, especially for identification of ultra-risk DLBCL patients. Dmax can be a promising indicator to evaluate the dissemination feature of lymphoma and incorporating dissemination feature can significantly improve the clinical management.

### Electronic supplementary material

Below is the link to the electronic supplementary material.

**Figure S1** Measurement of MBV. MBV (cm3) was defined as the product of the cross-sectional area of the larger section of the largest lesion and section thickness. MBV, metabolic bulk volume. **Table S1** Multivariable analysis of PFS and OS of DLBCL patient considering Dmax and MBV as continuous values. **Table S2** Spearman correlation analysis of Dmax and MBA groups. **Table S3** Multivariable analysis of PFS and OS of DLBCL patients staged based on Lugano System. **Table S4** Multivariable analysis of IPI and Dmax

## Data Availability

The datasets generated and/or analysed during the current study are not publicly available due being generated based on information collected during clinical care but are available from the corresponding author on reasonable request.

## Abbreviations

DLBCL: Diffuse large B cell lymphoma
NHL: Non-Hodgkin lymphoma
RCHOP: Rituximab, cyclophosphamide, doxorubicin, vincristine, and prednisone
OS: Overall survival
IPI: International prognostic Index
ECOG PS: Eastern Cooperative Oncology Group performance status
LDH: Lactate dehydrogenase
PFS: Progression-free survival
^18^F-FDG PET/CT: ^18^F-labeled fluorodeoxyglucose positron emission tomography with computed tomography
MTV: Metabolic tumor volume
HL: Hodgkin lymphoma
TTP: Time to progression
MBV: Metabolic bulk volume
IQR: Interquartile range
COO: Cell-of-origin
DRPI: Dmax revised prognostic index
HGAL: Human germinal center-associated lymphoma
AUC: Area under the curve
CI: Confidence interval
HR: Hazard ratio
PTCL: Peripheral T-cell Lymphomas
AITL: Angioimmunoblastic T-cell lymphoma
