# Supplementary material for: Dissemination feature based on PET/CT is a risk factor for diffuse large B cell lymphoma patients outcome
Source: BMC Cancer. 2023 Nov 29;23:1165. doi: 10.1186/s12885-023-11333-z (PMC10687880; doi:10.1186/s12885-023-11333-z)
Supplement: Supplementary file 1 — Figure S1 Measurement of MBV. MBV (cm3) was defined as the product of the cross-sectional area of the larger section of the largest lesion and section thickness. MBV, metabolic bulk volume. Table S1 Multivariable analysis of PFS and OS of DLBCL patient considering Dmax and MBV as continuous values. Table S2 Spearman correlation analysis of Dmax and MBA groups. Table S3 Multivariable analysis of PFS and OS of DLBCL patients staged based on Lugano System. Table S4 Multivariable analysis of IPI and Dmax [file 12885_2023_11333_MOESM1_ESM.docx]

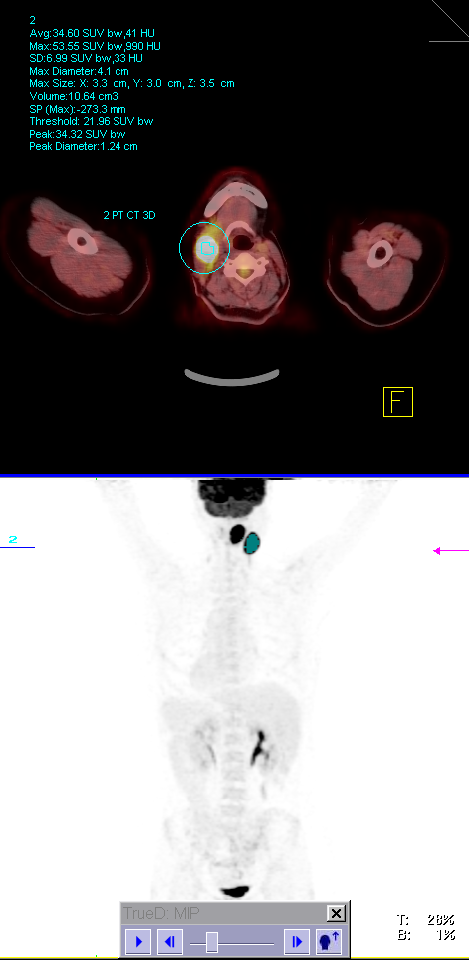


**Figure S1** Measurement of MBV. MBV (cm^3^) was defined as the product of the cross-sectional area of the larger section of the largest lesion and section thickness.

MBV, metabolic bulk volume.

**Table S1** Multivariable analysis of PFS and OS of DLBCL patient considering Dmax and MBV as continuous values.

| Variables |  | PFS |  |  |  | OS |  |
| --- | --- | --- | --- | --- | --- | --- | --- |
|  | HR | 95%CI | *p* value |  | HR | 95%CI | *p* value |
| Dmax | 1.011 | 1.001-1.022 | 0.026 |  | 1.016 | 1.003-1.029 | 0.017 |
| MBV | 1.001 | 0.999-1.003 | 0.160 |  | 1.002 | 1.000-1.005 | 0.029 |
| Age | 1.004 | 0.987-1.021 | 0.650 |  | 1.027 | 1.004-1.052 | 0.024 |
| Advanced Stage (Ann Arbor) | 2.151 | 1.088-4.251 | 0.028 |  | 2.192 | 0.908-5.292 | 0.081 |
| Extranodal sites > 1 | 0.987 | 0.619-1.574 | 0.958 |  | 0.654 | 0.373-1.146 | 0.138 |
| LDH >250 | 0.849 | 0.505-1.428 | 0.538 |  | 1.031 | 0.542-1.962 | 0.925 |
| ECOG PS ≥ 2 | 1.658 | 1.052-2.614 | 0.029 |  | 2.086 | 1.204-3.613 | 0.009 |
| Bulky (≥ 7.5cm) | 1.086 | 0.641-1.842 | 0.759 |  | 0.988 | 0.530-1.842 | 0.969 |

CI, confidence interval; ECOG PS, Eastern Cooperative Oncology Group performance status; HR, Hazard Rate; MBV, metabolic bulk volume; LDH, serum lactate dehydrogenase; OS, overall survival; PFS, progression-free survival.

**Table S2** Spearman correlation analysis of Dmax and MBA groups.

| Correlation of Dmax and MBV | |
| --- | --- |
| Correlation coefficient | 0.238 |
| p-value | <0.001 |

MBV, metabolic bulk volume.

**Table S3** Multivariable analysis of PFS and OS of DLBCL patients staged based on Lugano System.

| Variables |  | PFS |  |  |  | OS |  |
| --- | --- | --- | --- | --- | --- | --- | --- |
|  | HR | 95%CI | *p* value |  | HR | 95%CI | *p* value |
| Dmax (> 45.34 cm) | 1.918 | 1.137-3.236 | 0.015 |  | 2.302 | 1.189-4.459 | 0.013 |
| MBV (> 21.65 cm^3^) | 1.486 | 0.913-2.419 | 0.111 |  | 2.209 | 1.212-4.025 | 0.010 |
| Age | 1.002 | 0.986-1.019 | 0.784 |  | 1.029 | 1.005-1.055 | 0.020 |
| Advanced stage  (Lugano Stage) | 1.783 | 0.858-3.705 | 0.121 |  | 1.570 | 0.609-4.046 | 0.351 |
| Extranodal sites > 1 | 1.086 | 0.680-1.735 | 0.730 |  | 0.749 | 0.428-1.310 | 0.311 |
| LDH >250 | 0.821 | 0.487-1.386 | 0.461 |  | 0.941 | 0.497-1.784 | 0.853 |
| ECOG PS ≥ 2 | 1.779 | 1.117-2.833 | 0.015 |  | 2.360 | 1.350-4.126 | 0.003 |
| Bulky | 1.155 | 0.698-1.914 | 0.575 |  | 1.040 | 0.0.575-1.882 | 0.896 |

CI, confidence interval; ECOG PS, Eastern Cooperative Oncology Group performance status; HR, Hazard Rate; MBV, metabolic bulk volume; LDH, serum lactate dehydrogenase; OS, overall survival; PFS, progression-free survival.

**Table S4** Multivariable analysis of IPI and Dmax.

| Variables |  | PFS |  |  |  | OS |  |
| --- | --- | --- | --- | --- | --- | --- | --- |
|  | HR | 95% CI | p-value |  | HR | 95% CI | p-value |
| Dmax (> 45.34cm) | 2.356 | 1.454-3.815 | <0.001 |  | 2.734 | 1.502-4.978 | 0.001 |
| IPI (≥ 3) | 1.741 | 1.068-2.838 | 0.026 |  | 1.998 | 1.086-3.675 | 0.026 |

CI, confidence interval; HR, Hazard Rate; IPI, International prognostic Index; OS, overall survival; PFS, progression-free survival.
